# Supplementary material for: Identification of Novel Sphydrofuran-Derived Derivatives with Lipid-Lowering Activity from the Active Crude Extracts of Nocardiopsis sp. ZHD001
Source: Int J Mol Sci. 2023 Feb 1;24(3):2822. doi: 10.3390/ijms24032822 (PMC9917766; doi:10.3390/ijms24032822)

Supplementary material for

Identification of Novel Sphydrofuran-Derived Derivatives with Lipid-Lowering Activity from the Active Crude Extracts of *Nocardiopsis* sp. ZHD001

Yuhong Tian [1], Yongjun Jiang [1,*], Zhengshun Wen [1], Liping Guan [1], Xiaokun Ouyang [1], Wanjing Ding[2] and Zhongjun Ma [2,*]

[1] School of Food and Pharmacy, Zhejiang Ocean University, Zhoushan 316022, People's Republic of China

[2] Institute of Marine Biology and Pharmacology, Ocean College, Zhejiang University, Zhoushan 316021, People's Republic of China

[*] Correspondence: jiangyj@zjou.edu.cn (Y.J.); Mazj@zju.edu.cn (Z.M.)

Contents	pages
**Figure S1.** Colonies of *Nocardiopsis* sp. ZHD001	**4**
**Figure S2.** Body weight of mice.	**4**
**Figure S3** HR-ESI-MS spectrum of compound **1**	**5**
**Figure S4.** IR spectrum of compound **1**	**5**
**Figure S5.** ^{1}H NMR spectrum of compound **1** in Methanol-d_4	**6**
**Figure S6.** ^{13}C NMR spectrum of compound **1** in Methanol-d_4	**6**
**Figure S7.** ^{1}H-^{1}H COSY spectrum of compound **1** in Methanol-d_4	**7**
**Figure S8.** HSQC spectrum of compound **1** in Methanol-d_4	**7**
**Figure S9.** HMBC spectrum of compound **1** in Methanol-d_4	**8**
**Figure S10.** HR-ESI-MS spectrum of compound **2**	**8**
**Figure S11.** IR spectrum of compound **2**	**9**
**Figure S12.** ^{1}H NMR spectrum of compound **2** in Methanol-d_4	**9**
**Figure S13.** ^{13}C NMR spectrum of compound **2** in Methanol-d_4	**10**
**Figure S14.** ^{1}H-^{1}H COSY spectrum of compound **2** in Methanol-d_4	**10**
**Figure S15.** HSQC spectrum of compound **2** in Methanol-d_4	**11**
**Figure S16.** HMBC spectrum of compound **2** in Methanol-d_4	**11**

**Figure S17.** HR-ESI-MS spectrum of compound **3**	**12**
**Figure S18.** ^{1}H NMR spectrum of compound **3** in Methanol-d_4	**12**
**Figure S19.** ^{13}C NMR spectrum of compound **3** in Methanol-d_4	**13**
**Figure S20.** Oil red O of HepG2 cells under 40x mirror.	**13**

Figure S1. Colonies of *Nocardiopsis* sp. ZHD001

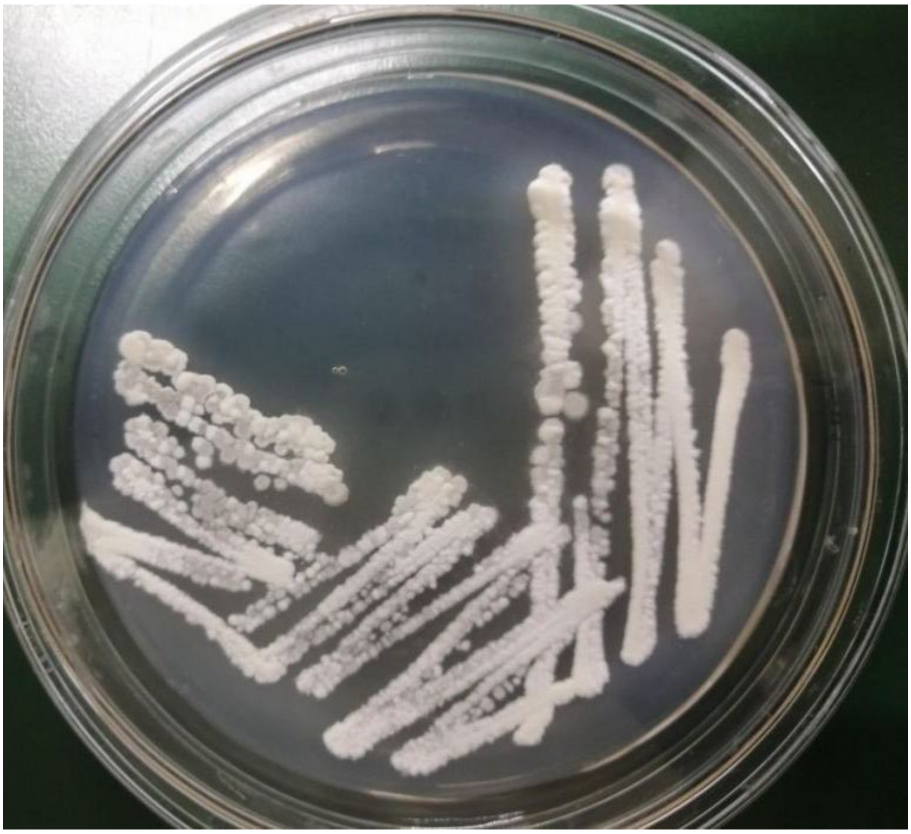

Figure S2. Body weight of mice.

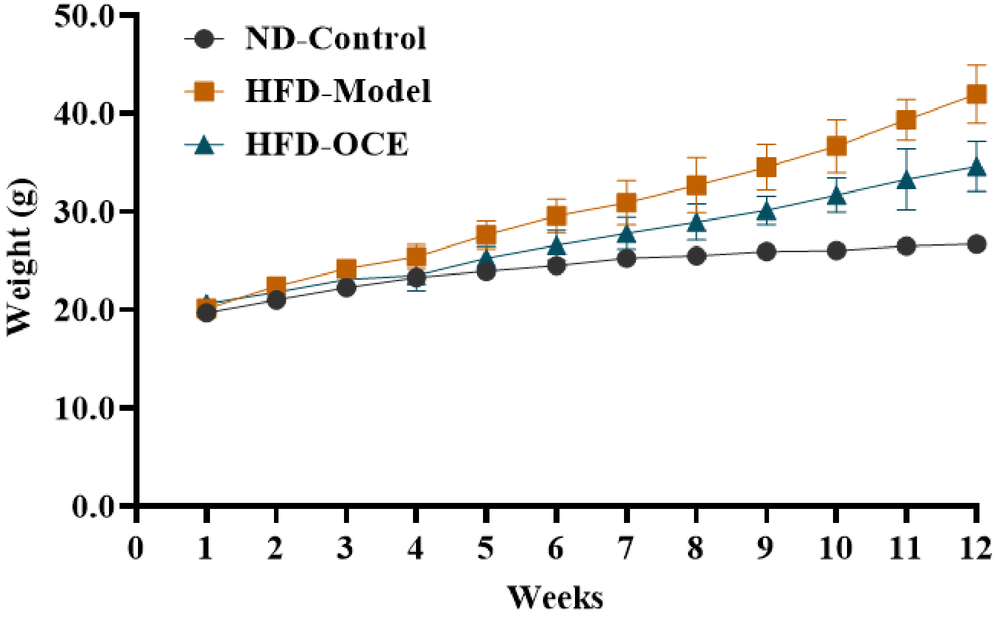

Figure S3. HR-ESI-MS spectrum of compound **1**

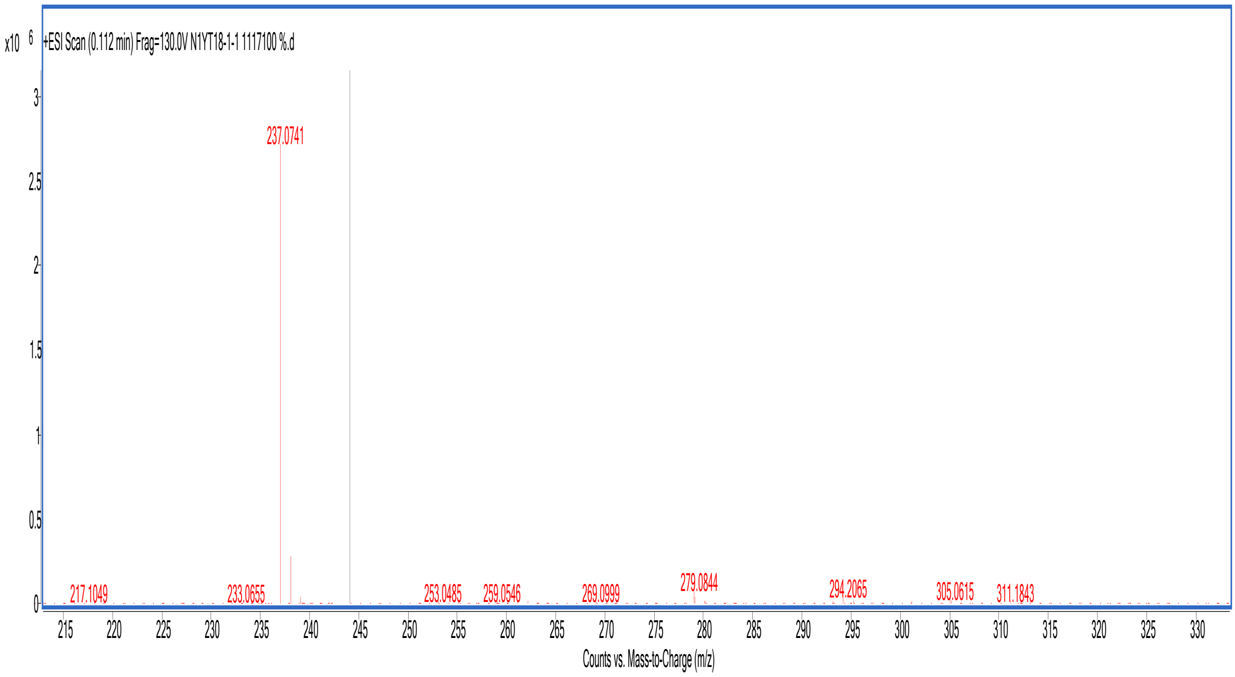

Figure S4. IR spectrum of compound **1**

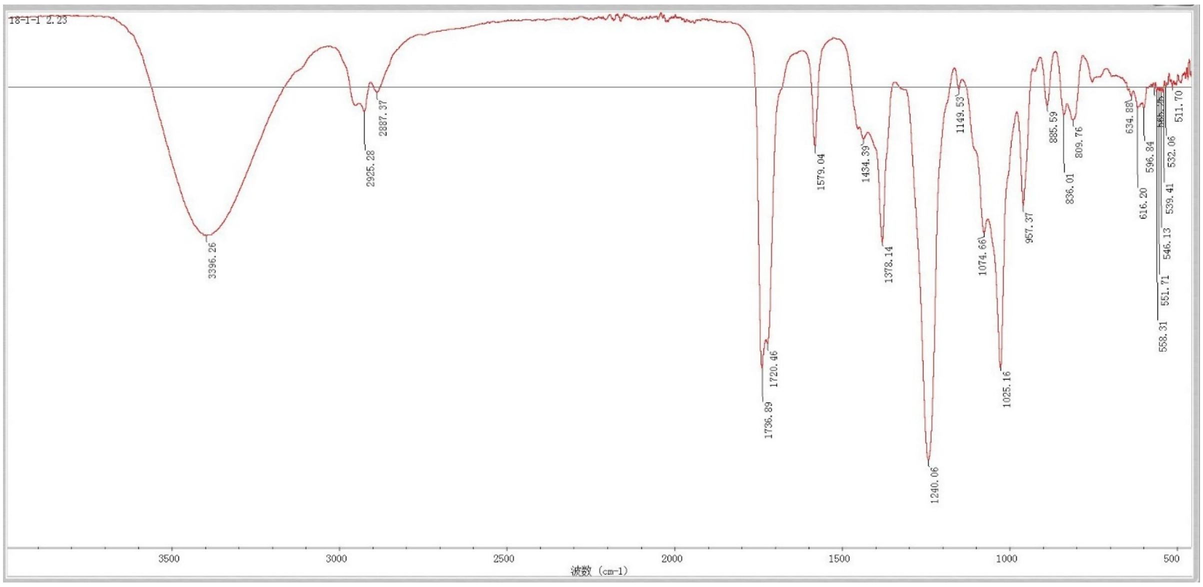

Figure S5. ^{1}H NMR spectrum of compound **1** in Methanol-d_4

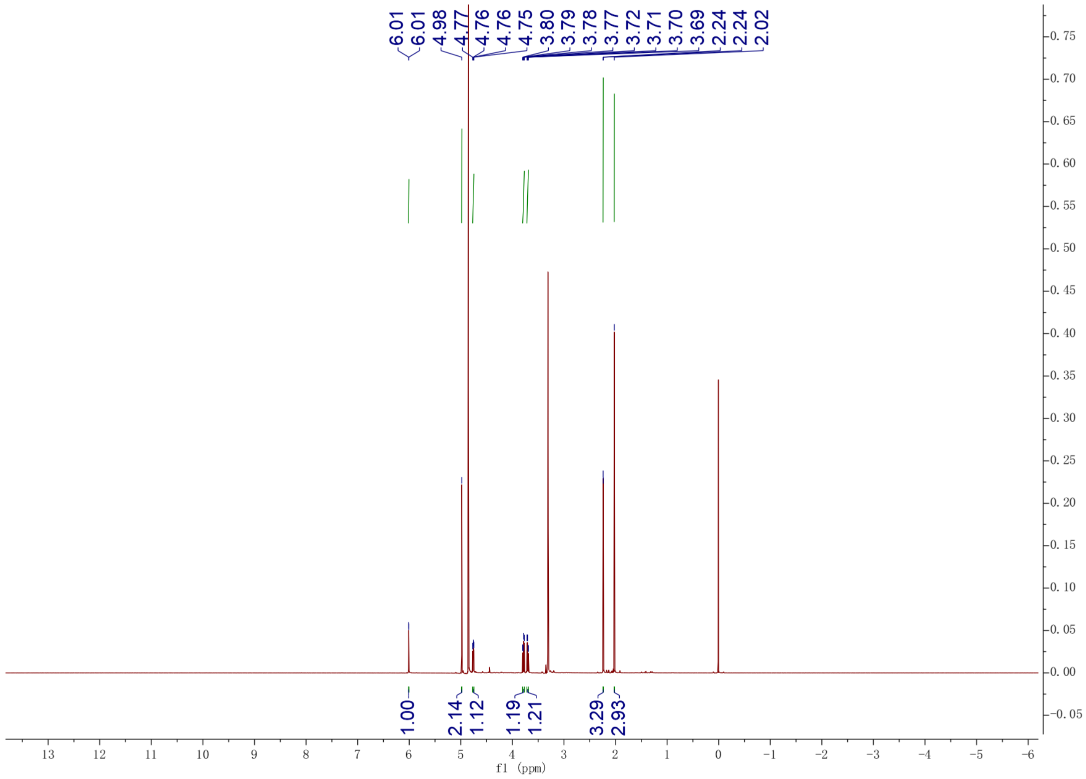

Figure S6. ^{13}C NMR spectrum of compound **1** in Methanol-d_4

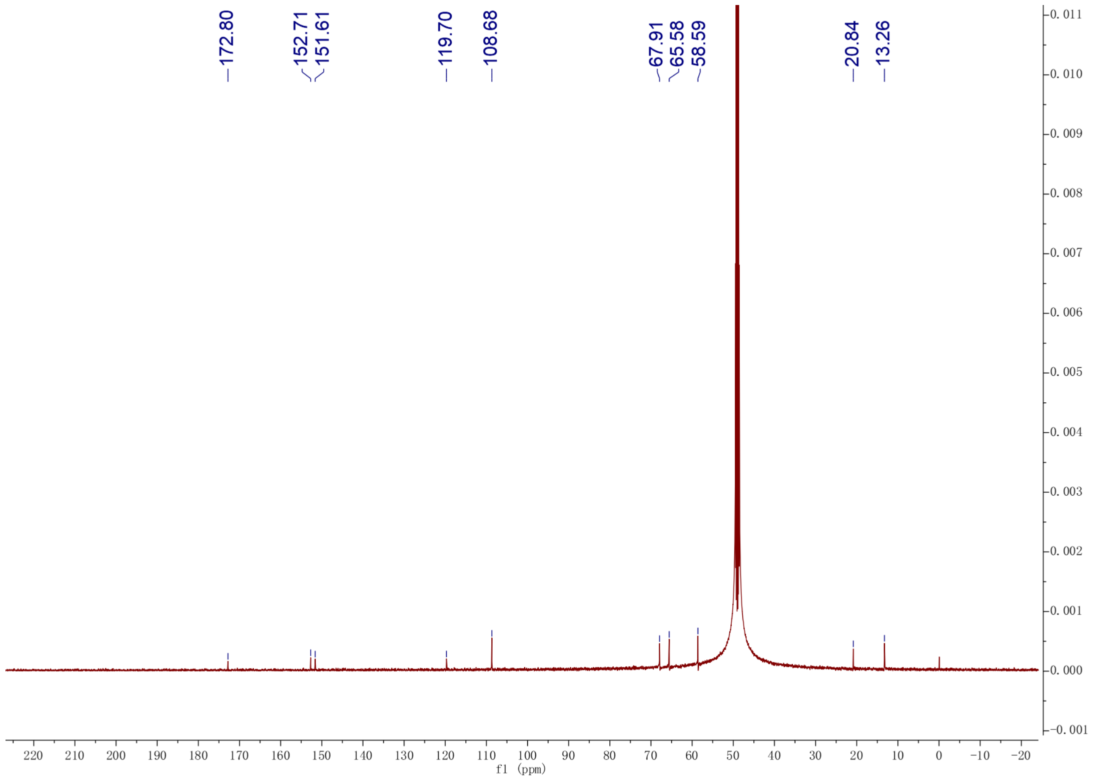

Figure S7. ^{1}H-^{1}H COSY spectrum of compound **1** in Methanol-d_4

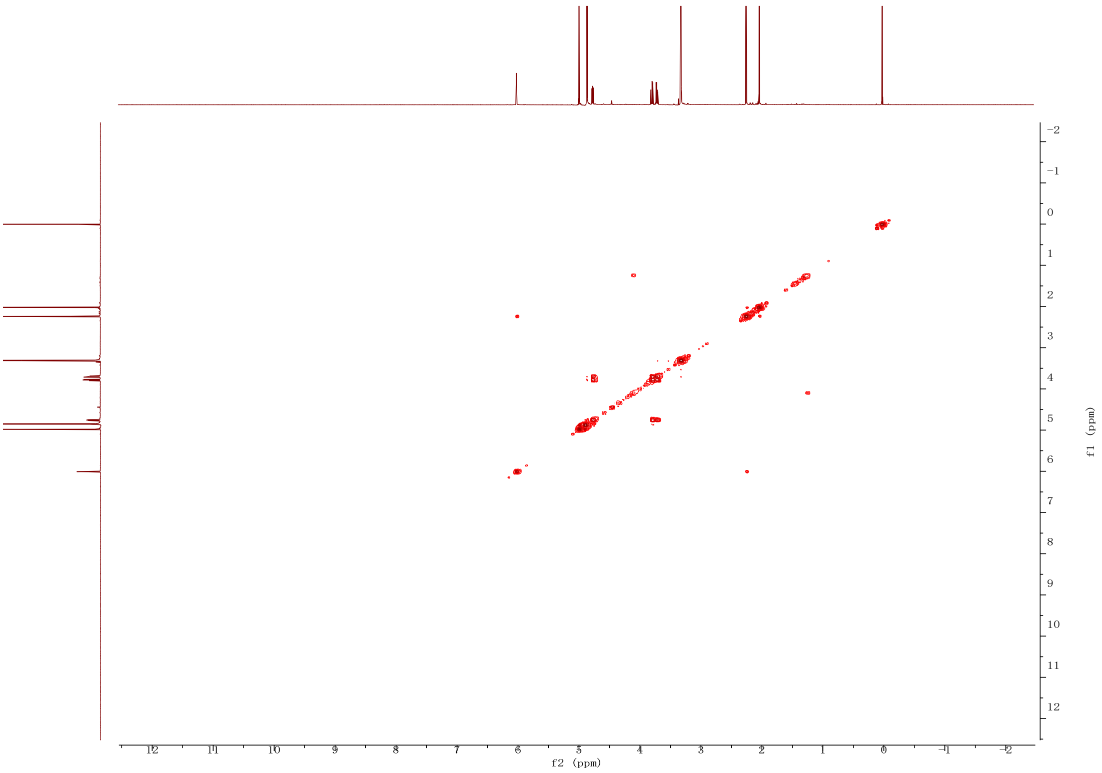

Figure S8. HSQC spectrum of compound **1** in Methanol-d_4

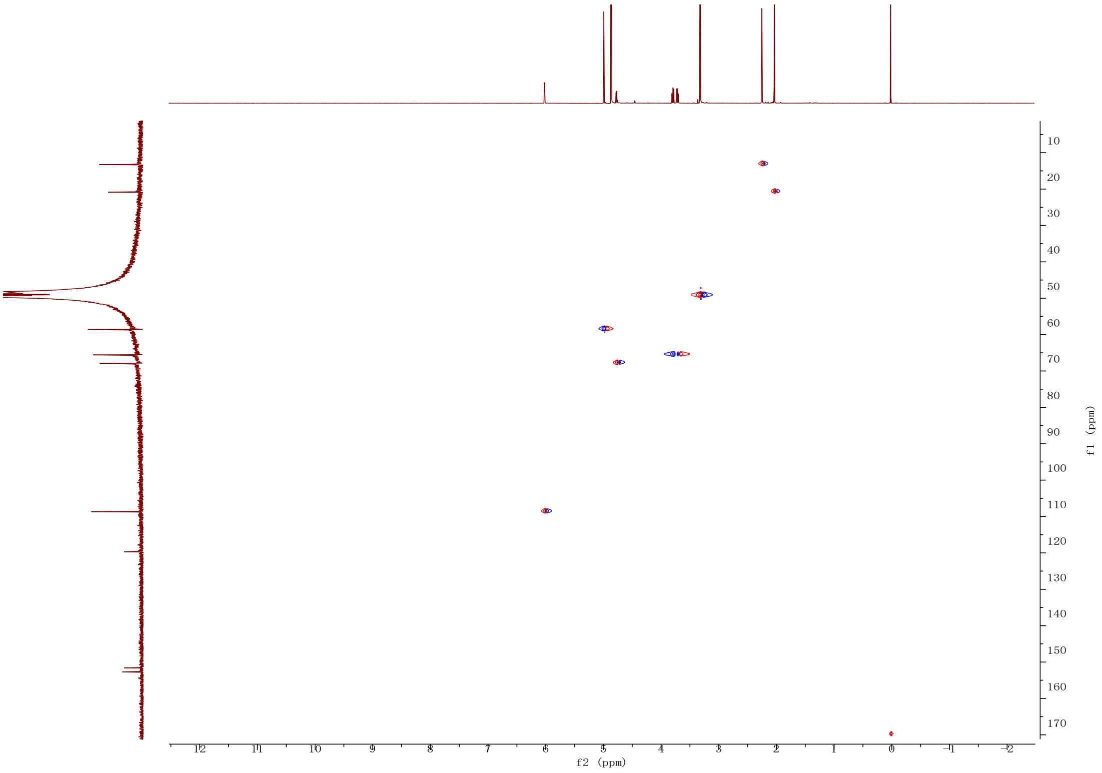

Figure S9. HMBC spectrum of compound **1** in Methanol-d_4

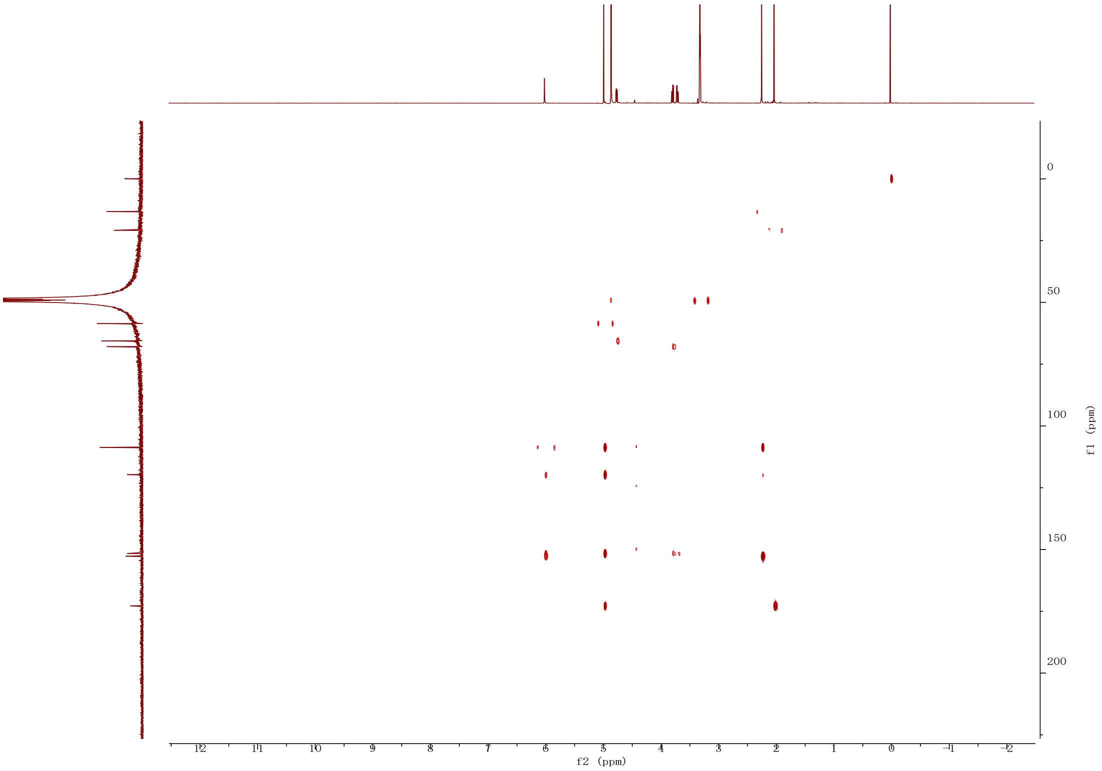

Figure S10. HR-ESI-MS spectrum of compound **2**

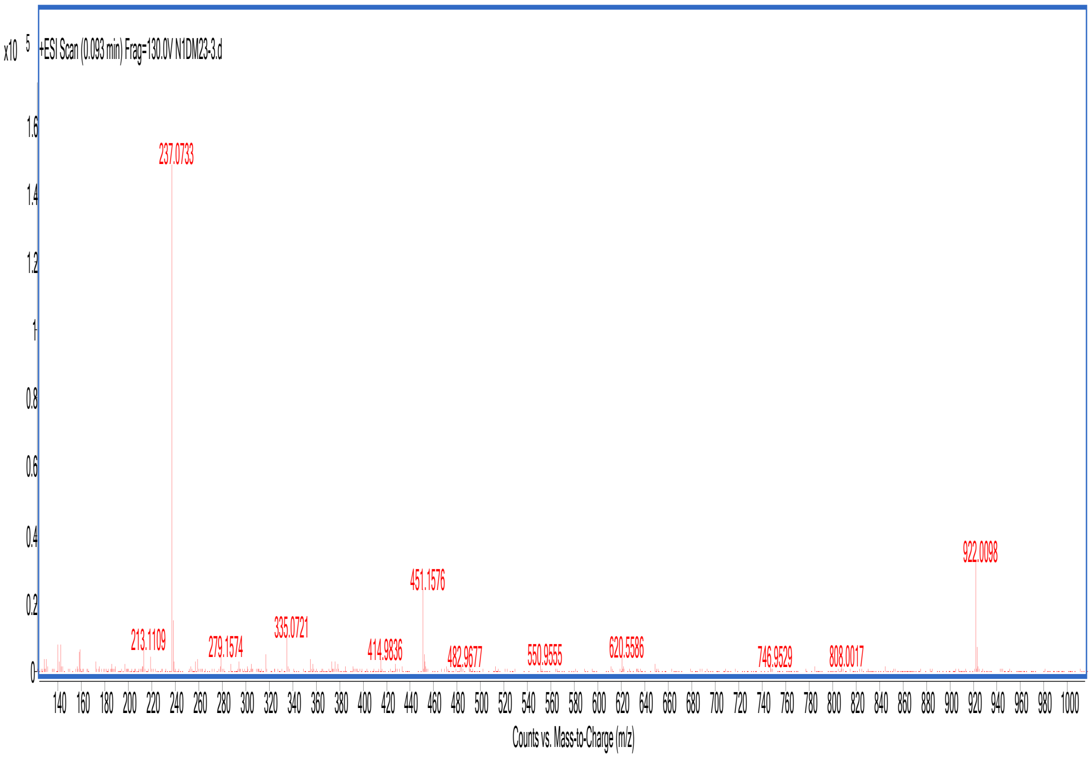

Figure S11. IR spectrum of compound **2**

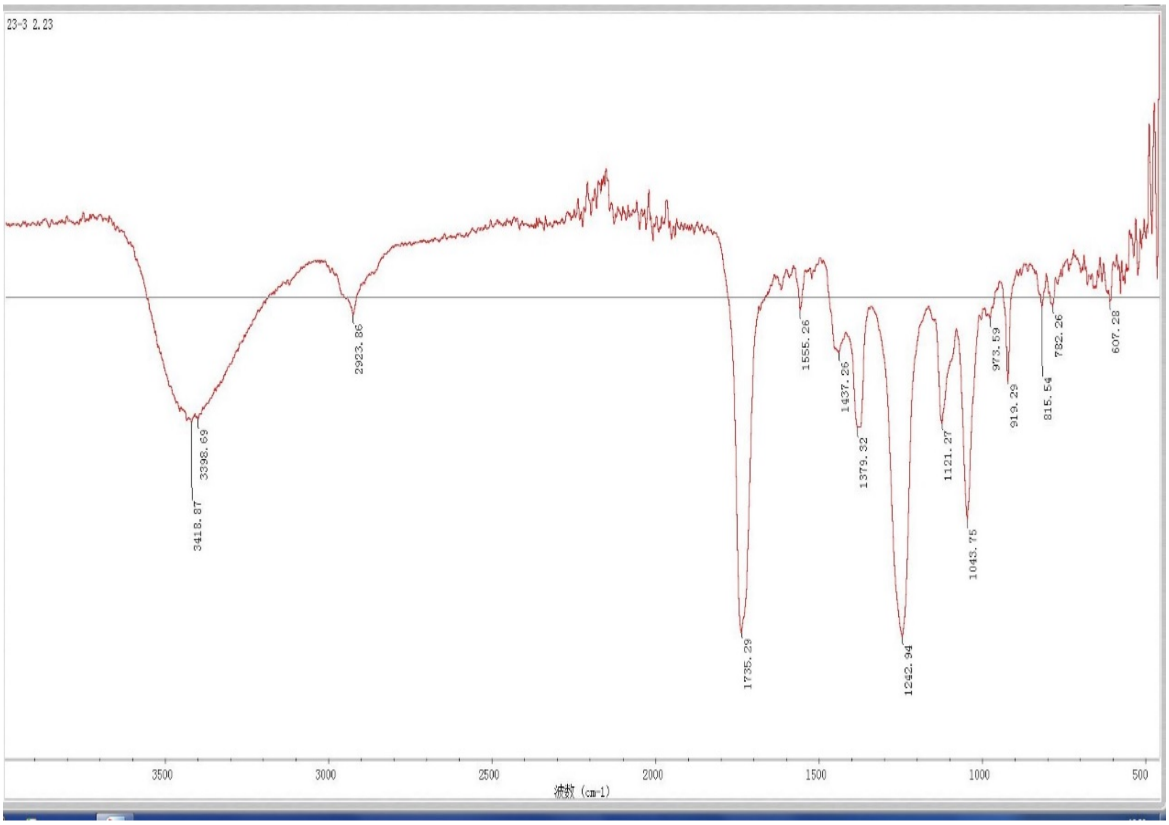

Figure S12. ^{1}H NMR spectrum of compound **2** in Methanol-d_4

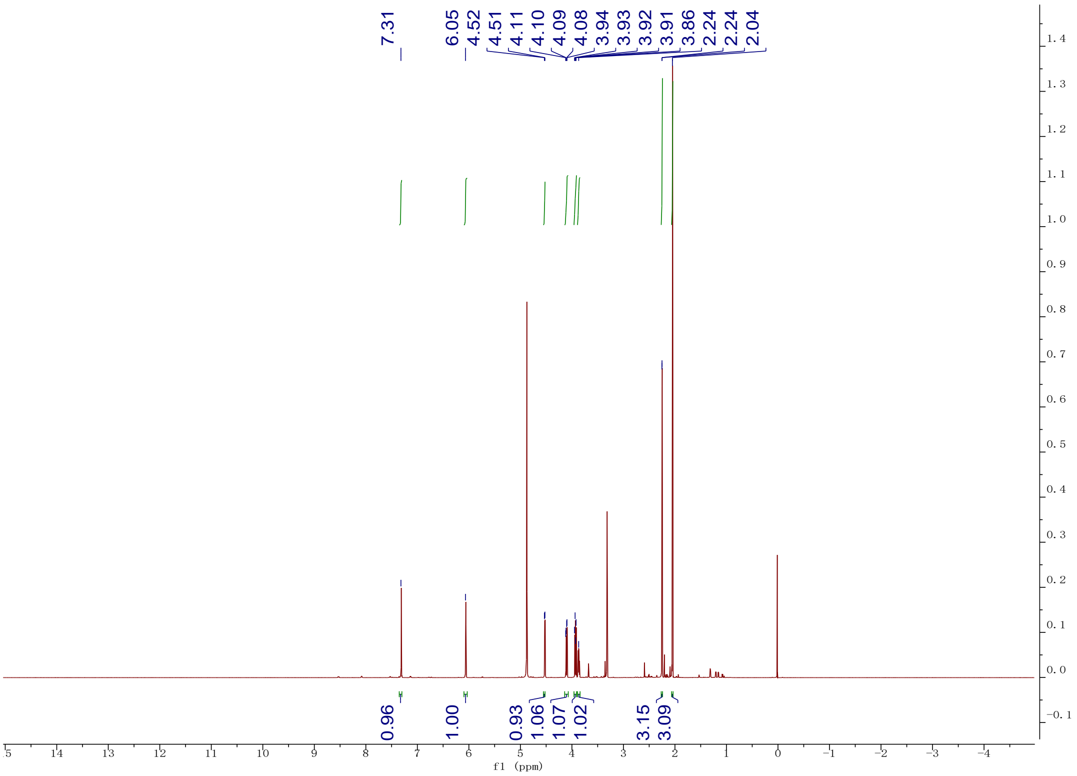

Figure S13. ^{13}C NMR spectrum of compound **2** in Methanol-d_4

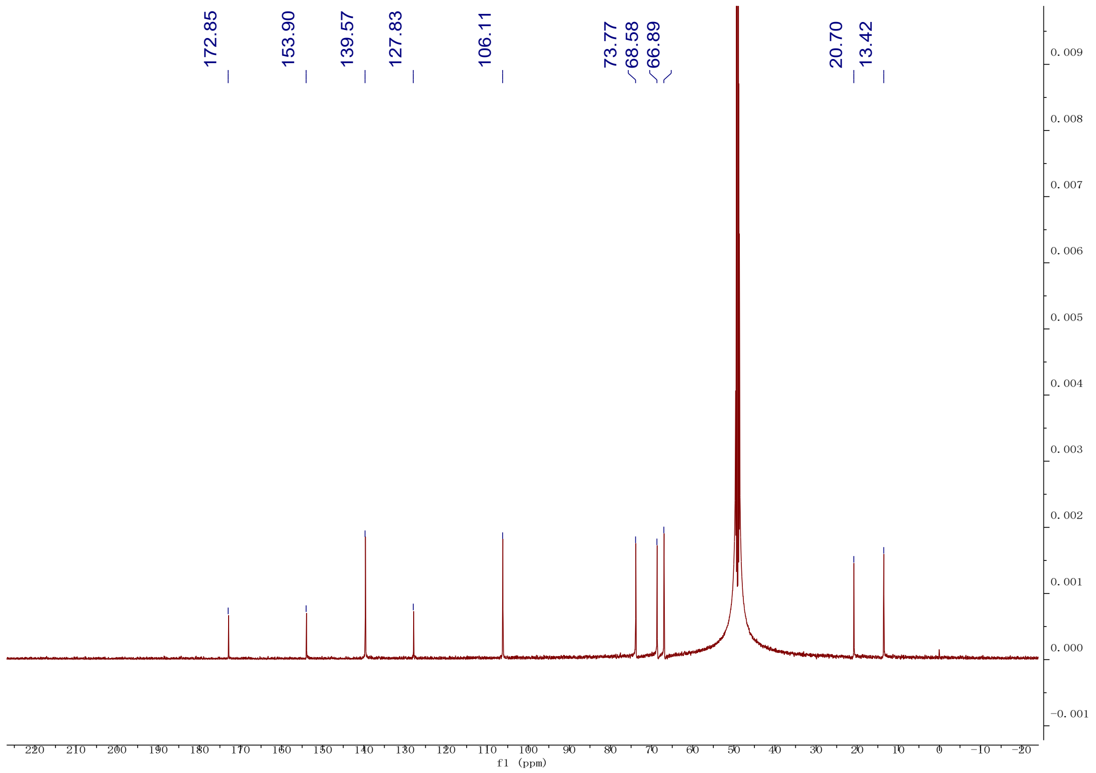

Figure S14. ^{1}H-^{1}H COSY spectrum of compound **2** in Methanol-d_4

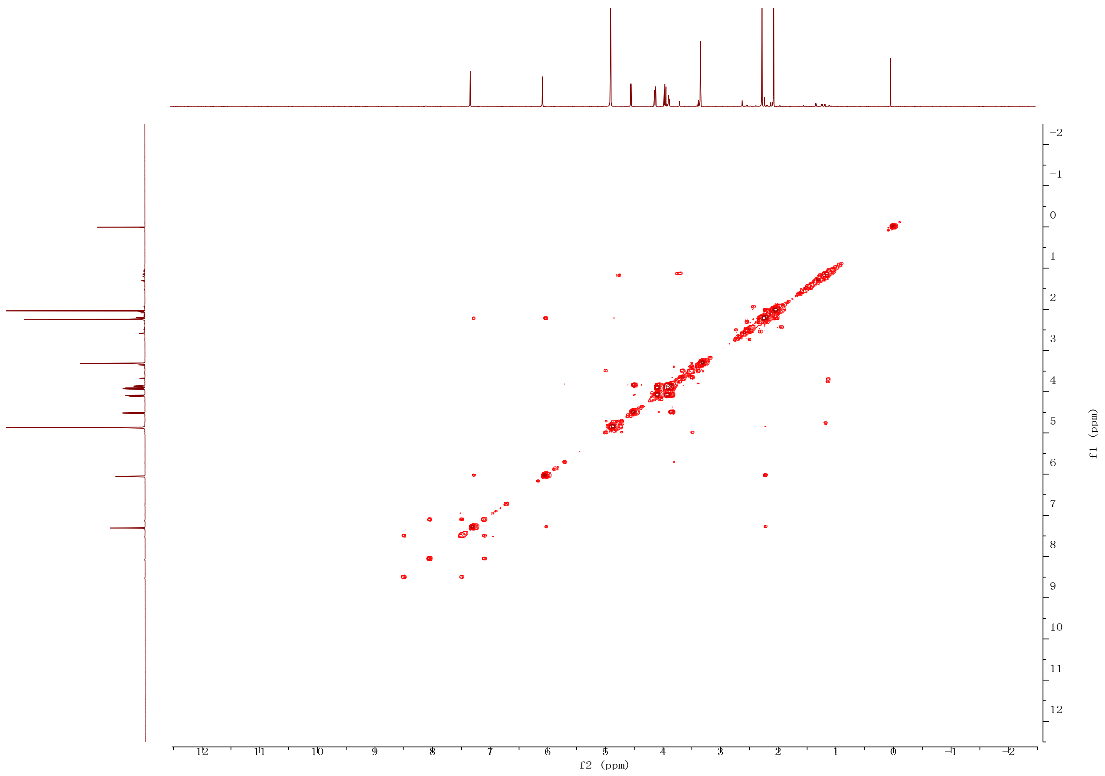

Figure S15. HSQC spectrum of compound **2** in Methanol-d_4

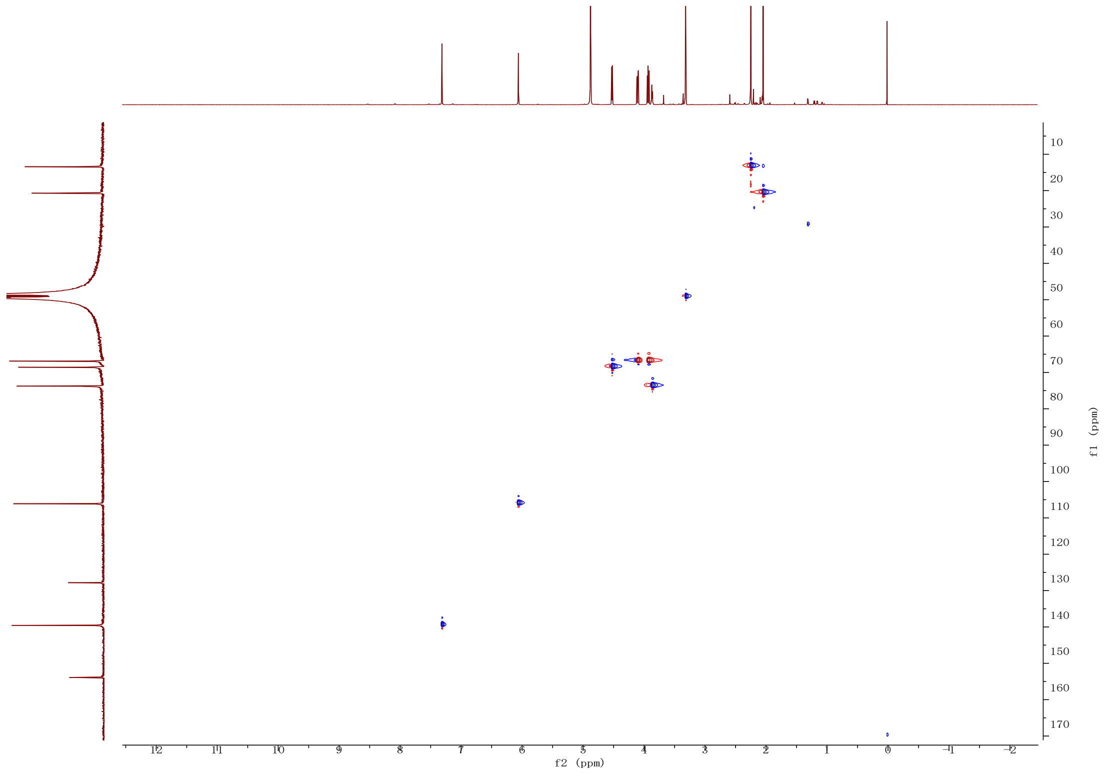

Figure S16. HMBC spectrum of compound **2** in Methanol-d_4

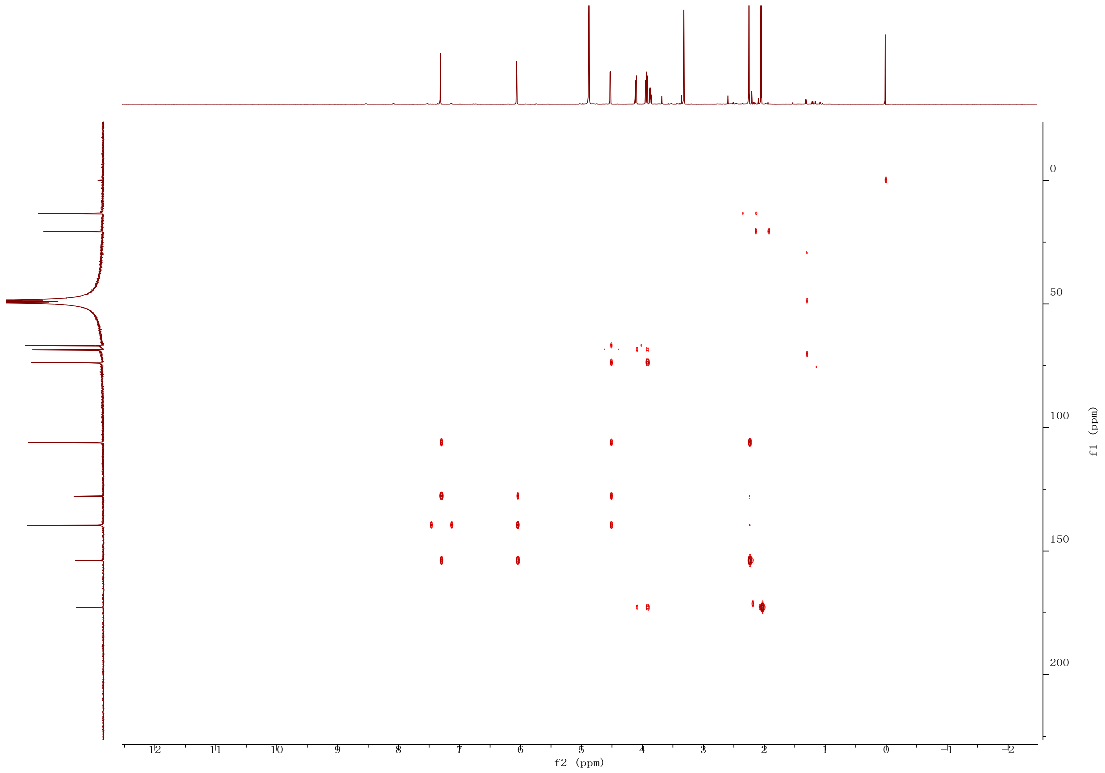

Figure S17. HR-ESI-MS spectrum of compound **3**

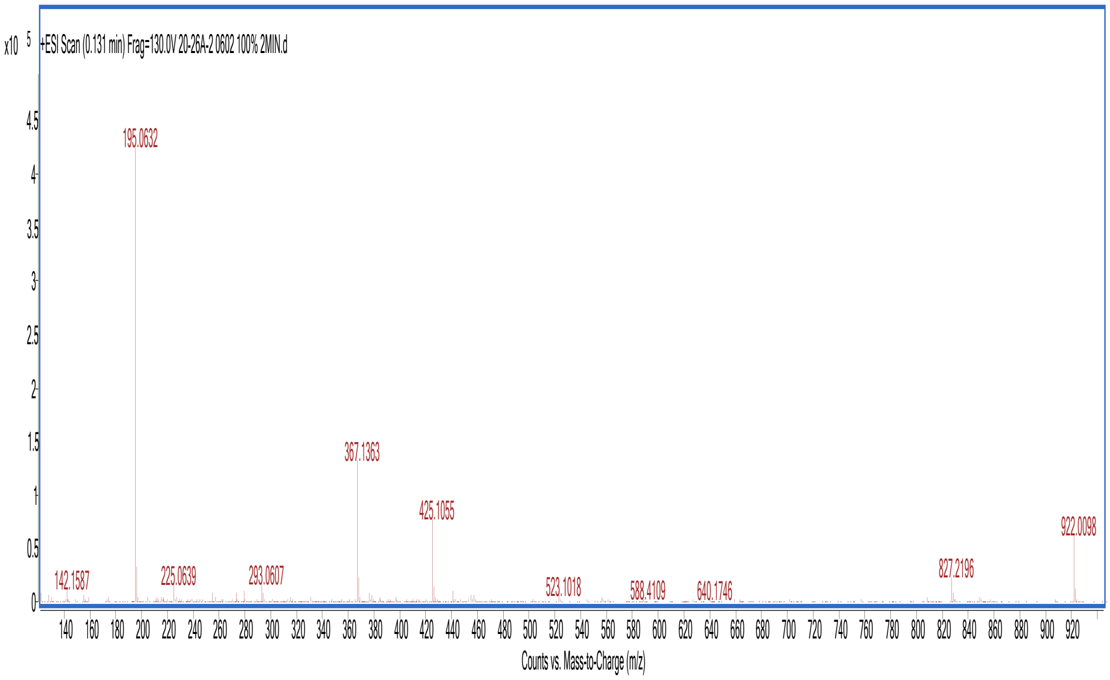

Figure S18. ^{1}H NMR spectrum of compound **3** in Methanol-d_4

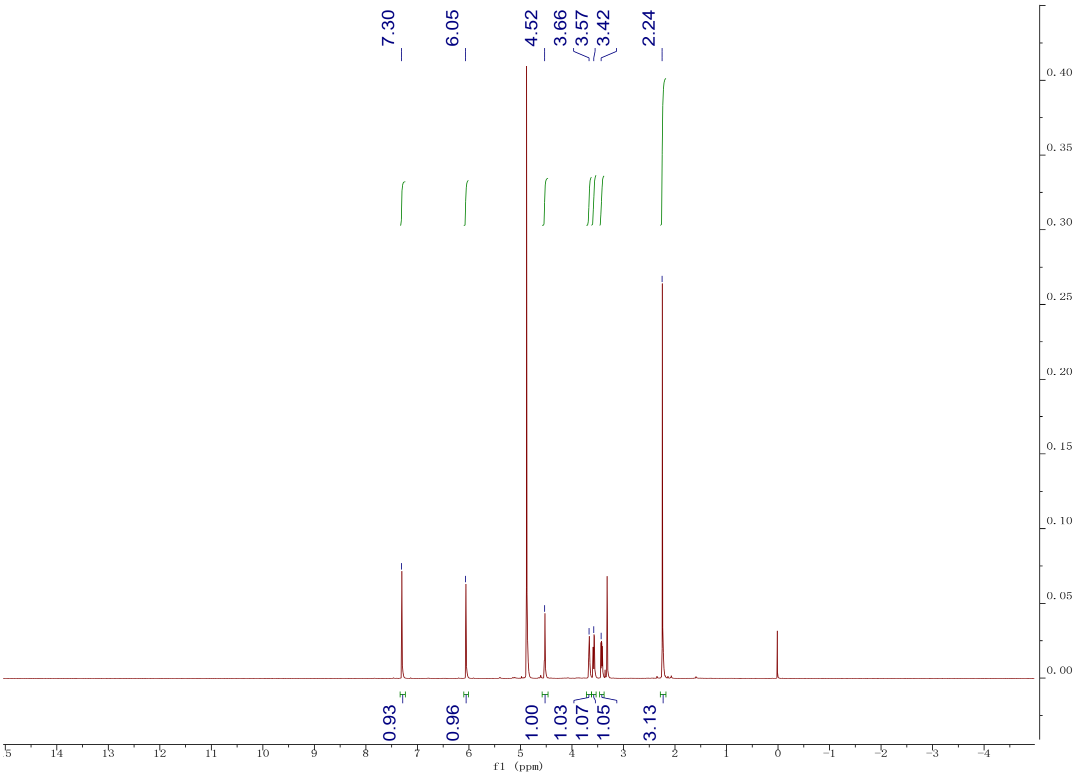

Figure S19. ^{13}C NMR spectrum of compound **3** in Methanol-d_4

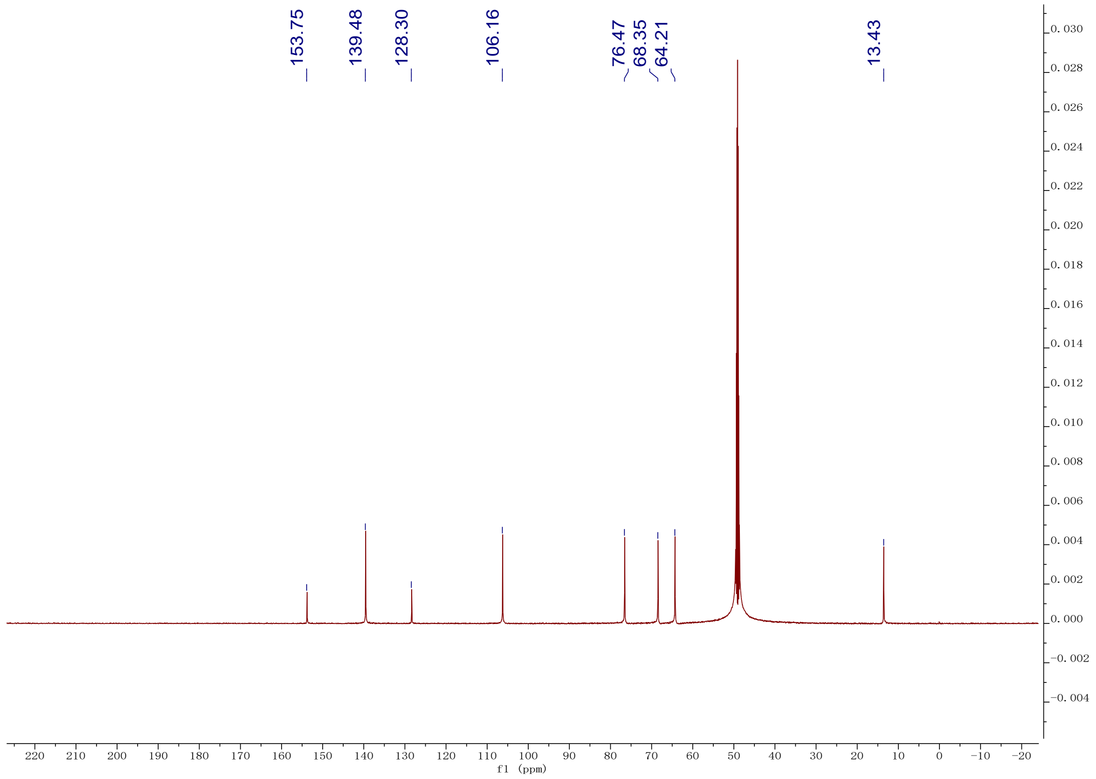

Figure S20. Oil red O of HepG2 cells under 40x mirror.

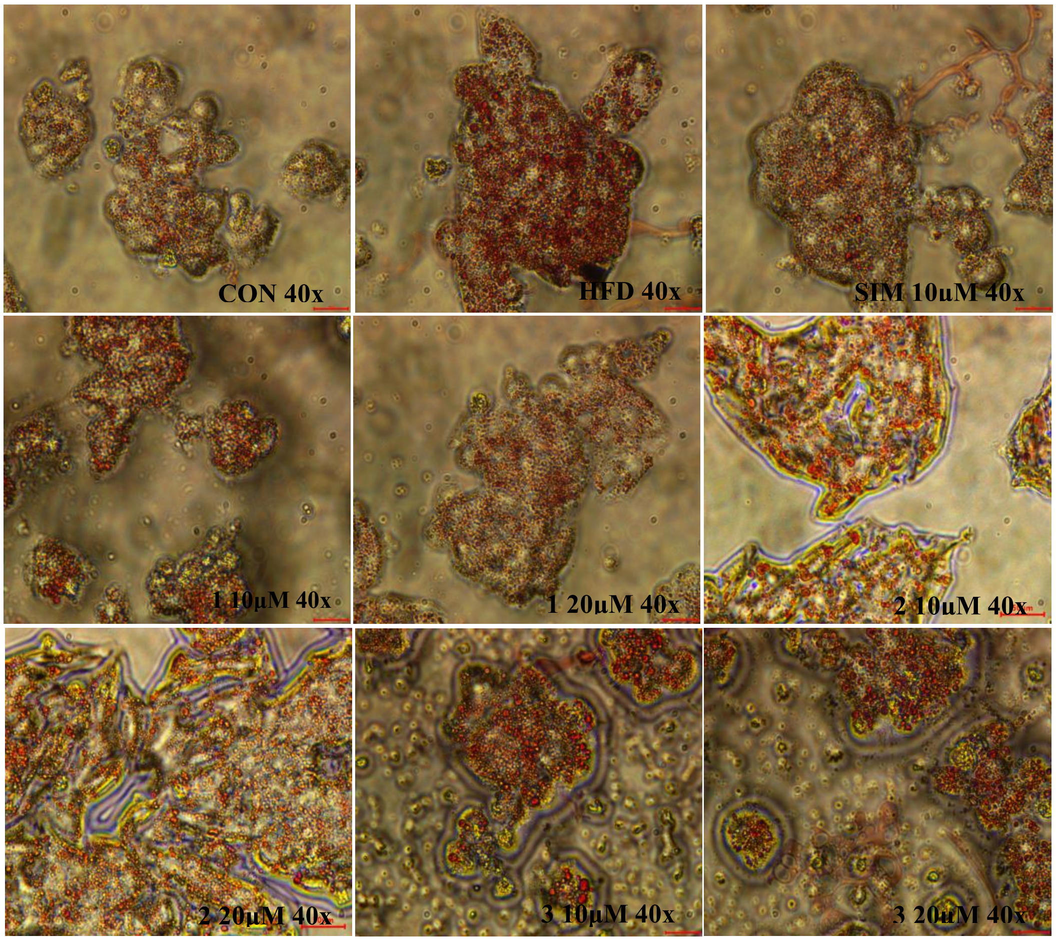

Supplement: Supplementary file 1 [file ijms-24-02822-s001.zip › ijms-2153745-supplementary.pdf]
